# COVID-19 and Beyond: Exploring Public Health Benefits from Non-Specific Effects of BCG Vaccination

**DOI:** 10.3390/microorganisms9102120

**Published:** 2021-10-09

**Authors:** Kent J. Koster, Hilary L. Webb, Jeffrey D. Cirillo

**Affiliations:** Department of Microbial Pathogenesis and Immunology, College of Medicine, Texas A&M University, 8447 Riverside Pkwy, Bryan, TX 77807, USA; kkoster@tamu.edu (K.J.K.); hilaryh2@gmail.com (H.L.W.)

**Keywords:** Bacille Calmette–Guérin, BCG, COVID-19, vaccine, public health policy

## Abstract

Bacille Calmette–Guérin (BCG) vaccination, widely used throughout the world to protect against infant tuberculous meningitis and miliary tuberculosis (TB), can provide broad non-specific protection against infectious respiratory diseases in certain groups. Interest in BCG has seen a resurgence within the scientific community as the mechanisms for non-specific protection have begun to be elucidated. The impact of the COVID-19 pandemic on nearly every aspect of society has profoundly illustrated the pressure that respiratory infections can place on a national healthcare system, further renewing interest in BCG vaccination as a public health policy to reduce the burden of those illnesses. However, the United States does not recommend BCG vaccination due to its variable effectiveness against adult TB, the relatively low risk of *Mycobacterium tuberculosis* infection in most of the United States, and the vaccine’s interference with tuberculin skin test reactivity that complicates TB screening. In this review, we explore the broad immune training effects of BCG vaccination and literature on the effects of BCG vaccination on COVID-19 spread, disease severity, and mortality. We further discuss barriers to scheduled BCG vaccination in the United States and how those barriers could potentially be overcome.

## 1. Overview

Bacille Calmette–Guérin (BCG) is a live attenuated vaccine derived from *Mycobacterium bovis* [1]. It is one of the most widely used vaccines globally, achieving 85% immunization among one-year-olds [2,3]. However, BCG vaccination policies in individual countries vary, ranging from universal infant vaccination to vaccination of “high risk” groups only, and some countries that formerly utilized BCG vaccination ceased vaccination once TB rates dropped [4]. The WHO currently recommends that in countries with a high burden of TB, a single dose of the BCG vaccine should be given to all infants as soon as possible after birth, even in areas with endemic HIV [3,5]. However, BCG vaccination is not a scheduled or recommended vaccine in the United States (U.S.), due to the low incidence of *Mycobacterium tuberculosis* (*Mtb*) infection in infants, a U.S. tuberculosis control strategy focused around early detection of *Mtb* infection and interruption of transmission, and extensive use of the low-cost tuberculin (purified protein derivative, PPD) skin test to screen for *Mtb* exposure or infection [6].

In 2020, COVID-19, caused by SARS-CoV-2, a novel coronavirus that originated in Wuhan, China, quickly spread into a global pandemic [7,8]. The World Health Organization (WHO) declared COVID-19 a pandemic on 11 March 2020. Lacking an effective vaccine capable of combatting this sudden global crisis, researchers examined potential strategies that were already available, and the BCG vaccine displayed significant potential and is already U.S. Food and Drug Administration (FDA) approved for treatment of bladder cancer [9]. Here, we review the history of the BCG vaccine, how its non-specific effects were found to protect from more than just tuberculosis (TB), and research into the mechanisms behind these protective effects. Literature covering BCG’s protective effects against pulmonary diseases and all-cause-mortality are reviewed, ultimately focusing on BCG’s potential as a tool against COVID-19. Finally, we specifically discuss considerations for implementing BCG vaccination in the U.S. as a tool for reducing the public health impacts of pandemics, similar to that caused by SARS-CoV-2.

## 2. History of the BCG Vaccine

Use of the BCG vaccine has sometimes been characterized by controversy and debate. The strain was originally isolated from the milk of a heifer identified to have tuberculous mastitis in 1901 and then transferred to Institut Pasteur in Lille, France [1,10]. In search of a vaccine for pulmonary TB, Léon Charles Albert Calmette and Jean-Marie Camille Guérin passaged their strain on potato slices soaked in ox bile and glycerol, after which they found it to be less virulent when injected into Guinea pigs [1,11]. Approximately 231 total passages, between 1908 to 1921, were conducted at intervals of every three weeks. The resulting bacterial strain was found to be immunogenic and protect immunized cattle against challenge from virulent *M. bovis* [12]. By the time lyophilization technology obviated the need to continually passage bacterial strains (1961), BCG had been passaged approximately 1173 times [13]. The strain was first used as a human vaccine against *Mtb* in 1921, and by 1924 approximately 660 infants had been orally vaccinated, and the strain became mass-produced in Lille and distributed to other laboratories internationally [1,10,11]. In France, 116,000 infants had been vaccinated by 1938, while in Germany BCG vaccination was abandoned in 1931 after 72 infants died the year prior from being injected with a contaminated vaccine. Fewer than 1000 vaccinations had been performed in the six years since BCG’s introduction into Germany [11].

Although BCG vaccination was widely accepted and promoted in Scandinavia, U.S. and British researchers were not convinced, pointing to a lack of randomized controlled trials, and inability to separate the effects of vaccination from those created by other anti-tuberculosis efforts such as improvements in general hygiene and better treatment [11]. While sanatorium therapy was the entrenched treatment philosophy in Britain, BCG vaccination regained momentum following a nursing shortage post-World War II. After being offered to nurses in 1949, a large trial on school children was commenced the following year, eventually resulting in the conclusion that BCG offered “substantial protection” [11]. Interestingly, vaccination of other school children was commenced even prior to the completion of that trial [11]. In the U.S., concerns over the safety of early BCG cultures, a focus on developing a heat-killed vaccine, and increased reliance on surgical intervention meant that BCG had few advocates. By the time that BCG vaccination became better accepted elsewhere, leaving the U.S. as the major hold-out nation, TB-control had shifted to an optimistic philosophy of wiping out TB through deployment of newly developed anti-tuberculosis drugs. A 1959 article in the British Medical Journal signed by 17 U.S. physicians spelled out their case against BCG vaccination, emphasizing a belief that detection of TB cases through tuberculin skin testing (TST) was essential to eradicating TB, and BCG vaccination’s conversion of subjects to TST positivity would interfere with this strategy [14].

This position is somewhat understandable, as BCG’s effectiveness at preventing TB infections has been complicated by variability in efficacy in different populations, requiring review of multiple studies to draw conclusions. A recent review of 24 randomized trials found reductions in TB rate ratios with BCG vaccination and the strongest results were from the studies that stringently used TST to exclude prior TB infection [15]. The study also concluded that protection against meningeal and miliary tuberculosis was quite good in infants and no significant difference in effect could be observed among the different strains of BCG. Providing a valuable long-term dataset, a follow-up study on American Indians and Alaska Natives who were part of a placebo-controlled BCG vaccine trial from 1935 to 1938 found an adjusted vaccine efficacy of 55%, reinforcing the study’s previously published 75% reduction in radiographically diagnosed tuberculosis at 11 years and 82% reduction in tuberculosis mortality at 20 years [16,17]. A 1994 meta-analysis covering 14 prospective trials and 12 case-control studies determined the risk ratio of TB to be 0.49 (CI95: 0.34–0.70) in BCG-vaccinated subjects and the odds-ratio from the case-control studies to be 0.50 (CI95: 0.39–0.64) [18]. The trials that examined tuberculous deaths showed BCG’s protective effect to be 71%, and those that examined meningitis showed a protective effect of 64%. Geographic latitude of the study site and the authors’ study validity score accounted for 66% of the heterogeneity among trials as observed in many reviews of BCG. Regardless of these seemingly positive results, part of the difficulty in assessing effectiveness of BCG is declining efficacy as the time since vaccination increases. Sterne et al. reviewed 10 randomized trials and found that efficacy decreased over time in seven of them, and increased in three [19]. Overall, the average efficacy a decade after BCG vaccination was 14% and, although their study was not comprehensive, they concluded from the trials examined that “There is no good evidence that BCG provides protection more than 10 years after vaccination”.

BCG has been shown to be effective even when administered to adolescents and young adults. Effectiveness of BCG vaccination at ages 15–24 years at preventing TB in England and Wales was 75%, with effectiveness of the vaccine remaining strong for at least a decade [20]. A 20-year evaluation of England’s policy of BCG vaccinating children ages 10–13 found a vaccine effectiveness of 84% during the first five years, averaging 77% over the entire study period, and gradually decreasing to similar incidence rates between BCG vaccinated and unvaccinated at 15–20 years, though incidence was low [21].

## 3. Non-Specific Effects: Improvements in Diverse Health Outcomes Renew Interest in BCG

Despite the U.S. governmental decision not to use scheduled BCG vaccination, interest in BCG vaccination’s non-specific effects continued. Cancer is one such area where BCG vaccination has been investigated as both a preventative and treatment. Old et al. found that BCG vaccination improved survival of mice challenged with tumor cells as far back as 1959 [22]. Interestingly, when studies suggested increased rates of lymphoma and leukemia in BCG-vaccinated populations, Usher et al. investigated by conducting a retrospective review of a placebo-controlled trial that included 2963 American Indian and Alaska Native schoolchildren younger than 20 years with no evidence of previous tuberculosis infection [23]. This 60-year follow-up found that although the overall rate of cancer diagnosis was not significantly different between groups, the rate of lung cancer was significantly lower in BCG recipients (18.2 vs. 45.4 cases per 100,000 person-years, representing a hazard ratio of 0.38 (CI95: 0.20–0.74)). BCG has been used extensively as a treatment for non-muscle-invasive bladder cancer and is introduced through intra-vesical instillation. It has been described as “one of the most successful biotherapies for cancer in use” thanks to demonstrating response rates of 55–70% in patients with high-risk stage I bladder cancer [24]. BCG’s toxicity has been low, and “to date it has not been surpassed by any other treatment” [25]. In a review of BCG for treatment of bladder cancer, five trials with recurrence information on 4767 patients were considered. 40.5% of the group receiving intra-vesical BCG therapy (with or without maintenance treatment, typically a three-week maintenance course at three months following the initial six applications) versus 49.7% of those without BCG had tumor recurrence [25,26]. Remarkably, neither chemotherapy nor BCG plus chemotherapy/immunotherapy were better than BCG alone, an effect which has also been experimentally observed [27].

Alzheimer’s disease and prevention of dementia is another area where beneficial effects from BCG have been observed. One retrospective study of bladder cancer patients found that patients who had been treated with BCG therapy benefited from a four-fold reduction in risk of Alzheimer’s disease relative to those not treated with BCG (hazard ratio 4.778 (CI95: 2.837–8.046), and saw no increase in prevalence of Parkinson’s disease [28]. Similarly, a large multi-center retrospective cohort study found that bladder cancer patients who received BCG therapy showed an Alzheimer’s disease hazard ratio of 0.726 (CI95: 0.529–0.996) when 75 years of age or older versus those not receiving BCG therapy [29]. Additionally, those receiving BCG showed a 28% reduction in risk of developing Parkinson’s disease.

One review on BCG vaccination and allergy found that BCG was unlikely to protect against allergic sensitization, but noted that the low quality of the reviewed studies meant that their conclusions should be “interpreted with caution” [30]. Despite a high degree of heterogeneity between studies, a more recent review of 16 epidemiological studies covering association between BCG vaccination and asthma found an overall pooled odds ratio of 0.86 (CI95: 0.79–0.93), supporting the hypothesis that BCG vaccination in early life reduces asthma [31].

Another area where BCG has been explored is in the reduction of diabetes. Mouse experiments suggested that BCG has protective effects against insulin dependent (type I) diabetes mellitus [32,33]. However, retrospective studies in humans have failed to conclusively demonstrate this connection [34]. More recently, an eight year prospective study of type I diabetic subjects with long-term disease receiving two doses of the BCG vaccine showed that, by the third year, BCG lowered hemoglobin A1c to near normal levels for the remainder of the study [35]. This effect was confirmed in a chemically diabetic mouse model. They found that BCG vaccination caused demethylation of T-regulatory cells’ signature tolerance genes that leads to enhanced mRNA expression.

## 4. Exploring the Mechanisms That Drive BCG’s Diverse Observed Effects

An excellent review of BCG immunomodulation is available that includes literature on BCG and COVID-19 [36]. Additionally, Butkeviciute, Jones, and Smith review mechanisms for heterologous effects of BCG vaccination, including changes in epigenetics, cytokine production, and antibody responses [37]. We provide a brief summary of the topic in Table 1. The concept that innate immunity can possess immunological memory of past insults has been coined “trained immunity” [38]. “Immune priming” by BCG vaccination may induce trained immunity that can assist with immune responses against infection by other bacteria, viruses, and fungi. This effect can be observed in studies showing increased cytokine responses in vitro following heterologous challenge after BCG vaccination, including in infants, where BCG may even accelerate development of the neonatal immune system [39,40,41,42]. Theoretically, elevated cytokine production should produce a rapid local immune response that results in efficient elimination of the pathogen before the infection can progress and induce higher levels of circulating cytokines [43]. These cytokines then stimulate CD4+ and CD8+ cells in an antigen-independent manner, referred to as “heterologous immunity” [36,44,45,46]. Epigenetic modifications, such as persistent histone modifications at promoters or enhancers, may drive the secondary trained immune response, since the epigenetic pathways involved are linked to key aspects of the cellular immune response [47,48].

Arts et al. investigated the mechanism for trained immunity due to BCG vaccination [43]. Genome-wide epigenetic reprograming of monocytes occurs—in particular, the H3K27ac histone mark produces an active chromatin state for promoters and enhancers. This had previously been identified in a study where epigenetic profiling of multiple targets (H3K4me1, H3K4me3, and H3K27ac), along with testing DNase I accessibility plus RNA sequencing, was performed prior to β-glucan priming and use of LPS to generate endotoxin-induced tolerance, and again at six days post-BCG vaccination [49]. The study confirmed that these marks represent phenotypic effects through the use of the attenuated yellow fever vaccine strain, finding significantly lower viremia in the BCG group. Interestingly, TNF-α and IL-6 levels post-BCG do not correlate with reduction in yellow fever viremia, though IL-1β does. In contrast with other studies, Th17 cytokines were not found to be predictive of viremia. Neutralizing yellow fever antibody titers also failed to correlate with cytokine induction. When exploring the epigenetic component of this response, the study identified that the NOD2 receptor was involved. The importance of the NOD2 receptor in induction of trained immunity by BCG has been demonstrated [40,50]. Monocytes from subjects with complete NOD2 deficiency failed to demonstrate trained immunity, while specific blockage of TLR2 or TLR4 receptors and the use of monocytes from a dectin-1-deficient individual all failed to prevent immune training [50]. When blood is collected from subjects before and after BCG vaccination, BCG increases nonspecific production of proinflammatory cytokines (IFNγ, TNF-α, and IL-1β) at two weeks and three months when PBMCs (peripheral blood mononuclear cells) are stimulated with killed *Mtb*, *Staphylococcus aureus*, and *Candida albicans* blastoconidia [51]. Significantly increased H3K4 trimethylation at cytokine and TLR4 promoters is observed after BCG vaccination, something that was previously associated with increased transcription of proinflammatory cytokine genes [52].

One important question is how epigenetically-mediated immune training can have a lasting effect when the lifespan of innate immune cells is limited [36,53]. Kaufmann et al. demonstrated that BCG “changes the transcriptional landscape” of long-lived hematopoietic stem cells in the bone marrow, and promotes polarization of multipotent progenitor cells towards myelopoiesis at the expense of lymphopoiesis [54]. They also found that macrophages derived from the bone marrows of mice that are BCG-vaccinated via the intravenous route provide significantly better protection against *Mtb* challenge than the PBS control, while those that were vaccinated via the subcutaneous route do not. The authors conclude “macrophages derived from the BM of BCG-iv vaccinated mice are intrinsically imprinted with an enhanced capacity to control the growth of *Mtb*.” The length of protection offered by the non-specific trained immunity effects of BCG is also of interest. Kleinnijenhuis et al. found that heterologous Th1 responses (measured by TNF-α production of PBMCs stimulated sonicated *Mtb* and heat-killed *Candida albicans* and *Staphylococcus aureus*) are strong at two weeks post-vaccination, and remain strong for a year for the two bacterial stimulants, with a similar pattern seen for Th17 responses [55]. However, TNF-α and IL-1β production effectively dropped to pre-vaccination levels one year after BCG vaccination.

Mathurin et al. found that resistance to vaccinia virus infection in mice vaccinated with BCG could be reduced by depleting CD4+ T cell populations, suggesting that both the innate and adaptive immune components play a role in trained immunity, and that cross-reactivity of epitopes may play a role [45]. Another study noted the similarity of T cell and B cell epitopes in the BCG Pasteur proteome with those in SARS-CoV-2 peptides [56]. They investigated the proteomes of other viruses against which BCG vaccination may provide protection, including influenza A, more respiratory and non-respiratory viruses, and the bacterium *Staphylococcus aureus,* along with the yeast *Candida albicans*. Using a threshold of 67% or greater identity with BCG sequences, the group found that the number of similar 9-mer and 15-mer peptide sequences correlated with the size of those proteomes. MHC class-I T cell restricted epitopes with greater than 67% similarity included the single NSP05 protein epitope, as well as the M pro chymotrypsin-like protease (a main protease involved in viral replication) and spike glycoprotein. However, no similar epitopes were found for MHC class-II-restricted T cell epitopes. When examining similar (62.5% or greater identity) B cell epitopes, numerous epitopes in structural proteins were identified, including eight in the spike glycoprotein, with one in the receptor binding domain where it could be targeted by neutralizing antibodies. BCG peptides have been experimentally demonstrated to induce a T cell response that is cross-reactive with SARS-CoV-2 [57]. When eight protein sequences with homology between BCG proteins and SARS-CoV-2 NSP3 and NSP13 proteins were utilized to stimulate T cells, perforin secretion following pulsing with corresponding SARS-CoV-2 antigens was enhanced for all 20 subjects’ CD8+ T cells. TNF, IFNγ, and IL-2 cytokine responses were significantly enhanced by seven of the eight tested peptides for all subjects’ ex vivo cultured CD4+ and CD8+ T cells, with the heterogeneity in responses being attributed to HLA differences. Blocking HLA removes this enhanced T cell cytokine response. Additionally, “bystander activation” of pre-existing effector or memory cells may also be induced by changes in the cytokine environment. The immune response to BCG can potentially stimulate production of cytokines or antibodies by existing effector T cells, reducing the threshold for activation of polyclonal effector T cells when challenged again by heterologous infections [36,58]. BCG may represent an ideal vaccine for inducing this bystander activation, as the TLR8 PAMP, a pathogen-associated molecular pattern detected by pattern-recognition receptors that are activated by bacterial RNA and play a key role in innate immunity, distinguishes between live and dead bacteria, regulating T_FH_ cell differentiation and downstream cytokine responses [59].

Non-specific effects of BCG may improve health of the immune system more generally by fighting “immune disequilibrium”. One model of immunity is that a healthy immune system is in a state of dynamic equilibrium between the internal environment and the surrounding microbial environment [60]. The effect of modern sanitation and medicine removing humans from the microbial environment in which their immune systems evolved could promote immune disequilibrium. An absence of stimulation involved in one type of immune response could lead to increases in other types of responses, leading to increased susceptibility to allergies or autoimmune diseases [61,62]. In fact, increases in food allergies have been noted in conjunction with “Westernization” [63]. This “hygiene hypothesis” could explain other beneficial effects of BCG [36]. It has certainly been noted that saprophytic mycobacteria can stimulate regulatory T cell responses, controlling eosinophil-mediated inflammation [64].

## 5. Effectiveness of BCG as a Vaccine beyond Those Known for Tuberculosis

With the greatest importance of BCG vaccination in areas of high endemic TB transmission, it is not unexpected that research sites in Africa have provided an abundance of data on the non-specific effects of BCG. A Ugandan study covering vaccination of infants with the Danish BCG strain at birth or six weeks after they were born was performed in 2014–2015 [65]. Subjects of both low and normal birthweight were followed for non-tuberculosis infectious disease rather than proxy measures such as hospital admission rates or all-cause mortality. In the first six weeks, the group receiving the vaccine displayed significantly lower incidence of non-tuberculosis infectious diseases (98 versus 129). In weeks 6–10 (when both groups had been vaccinated), cumulative incidence rates were not significantly different. The strongest effects were seen in boys, and infants with low birthweight. The mechanisms involved in the effects of BCG vaccination were dissected by looking for epigenetic changes. Histone trimethylation in PBMCs during the first six weeks is inhibited at several proinflammatory cytokine loci, including the TNF, IL-6, and IL-1β promoters. Surprisingly, no significant effect was seen on ex vivo production of TNF, IL-6, IL-1β, IL-10, and IFNγ following stimulation. A community-based case–control study in Guinea-Bissau found that infants with acute lower respiratory tract infections display an odds-ratio of being non-BCG vaccinated of 2.87 (CI95: 1.31–6.32), with the effect being greater in girls (4.45, CI95: 1.48–13.4) than in boys (1.72, CI95: 0.48–6.19) [66]. A similar retrospective epidemiological study in Europe used the Official Spanish Registry of Hospitalizations’ records of Basque Country, where neonatal BCG is part of the immunization schedule. This study found that hospitalization rates due to non-TB respiratory infections were significantly lower in BCG-vaccinated children, identifying a total preventive fraction of 41.4% [67].

The non-specific beneficial effects of BCG vaccination have been demonstrated in randomized, controlled trials. A trial on 105 low-birth-weight infants in Guinea-Bissau found that, when vaccinated with BCG at birth or first healthcare contact, the mortality rate ratio versus delayed vaccination was 0.17 (CI95: 0.02–1.35) within three days, 0.28 (0.06–1.37) in the first month, and 0.27 (0.07–0.98) after two months of age [68]. The significance decreased with age, which may be due to more subjects in the delayed-vaccination control group receiving their BCG vaccines, typically received at one month. However, a trial of 2320 low-birth-weight children found that at 12 months, the mortality rate ratio for early BCG versus delayed BCG controls was 0.83 (CI95: 0.63–1.08), which equates to a non-significant 17% reduction in infant mortality. The reduction was linked to less neonatal sepsis and fewer respiratory infections [69]. Both of these studies found that the benefit of early BCG vaccination was stronger in boys than in girls. Note that these studies compared early BCG vaccination to delayed vaccination, rather than no BCG vaccination at all, which likely controls for most variables associated with vaccination itself.

The beneficial effects of BCG vaccination are not limited to infants. In one placebo-controlled study, 60–75-year-old subjects were vaccinated once a month for three months in succession [70]. The study found increased IFNγ and IL-10 levels in the BCG group compared to the placebo group, and found that acute upper respiratory tract infections were significantly decreased. The Phase III ACTIVATE trial provided 198 elderly subjects with either BCG vaccination or a placebo, and followed them for twelve months [71]. Their interim results have been very promising. BCG vaccination significantly increased the time to first infection from a median of 11 weeks for the placebo group to 16 weeks for the BCG group. Furthermore, the incidence of new infections for the BCG group was 25.0% versus 42.3% for the placebo group, with the greatest protection being against respiratory tract infections of probable viral origin. The frequency of adverse events was not substantially different between groups, suggesting that BCG vaccination is both safe and effective for elderly recipients.

Pre-sensitization from environmental mycobacteria may reduce the effectiveness of BCG or possibly reduce the need for BCG in the lower latitudes, an observation suggested previously [72]. While it is intuitive to think that environmental mycobacteria exposure simply obviates the need for BCG to be used, with BCG providing little more effect than environmental mycobacteria might already provide, we surmise that it is unlikely that infants, who receive the most pronounced benefit from BCG vaccination, have been extensively exposed to environmental mycobacteria. Surprisingly, however, oral doses of *Mycobacterium avium* impaired their ability to control bacterial load following *Mtb* challenge, suggesting that the impact of environmental mycobacteria is more complicated than just any type of exposure to a single strain [73]. A Danish study of 4262 children found no statistically significant difference in the number of infectious illnesses from birth to 3 months, or from 3 to 13 months, resulting from BCG vaccination [74]. However, the authors observed a 38% reduction in the number of infectious illnesses in the first three months of life if the mother had previously received a BCG vaccine. Taken together, these observations suggest that BCG can have some beneficial effect in reducing disease, particularly respiratory infections, but that effect may not be the same in all populations.

## 6. BCG and COVID-19: A Strategy to Reduce the Public Health Burden of Epidemic Respiratory Disease?

**Figure 1:**
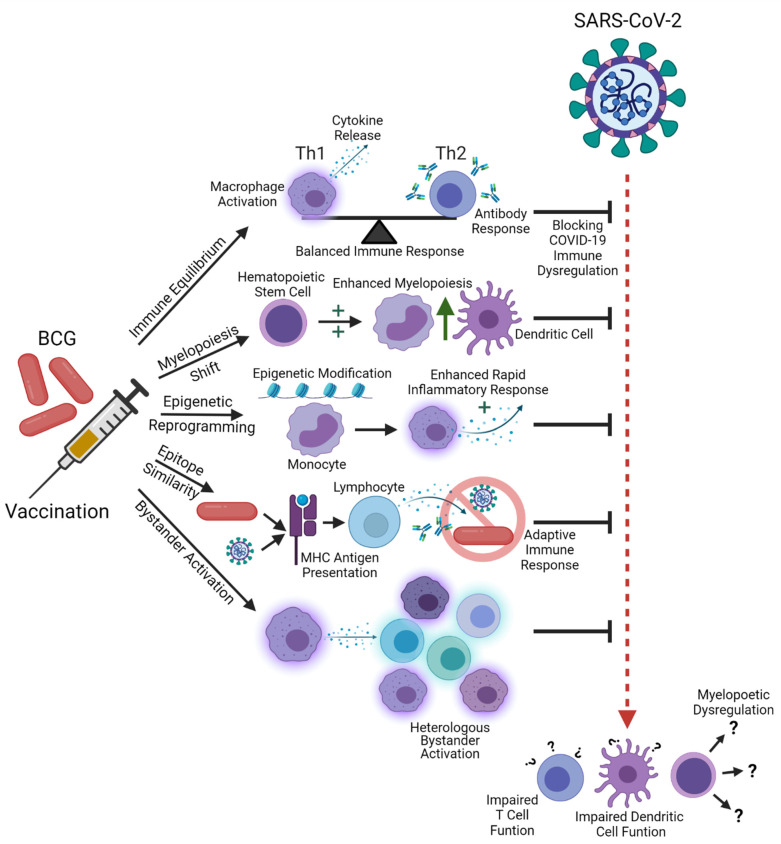
Effects of BCG’s non-specific immune-priming may collectively contribute to protection against COVID-19 and other severe infections. BCG vaccination has been shown to produce diverse effects on the immune system that can enhance its ability to defend against severe respiratory diseases. BCGs promotion of balanced “immune equilibrium” may fight pathogens’ induction of immune disequilibrium as an evasion strategy. The shift in bone marrow activity towards myelopoiesis induced by BCG vaccination could aid in resisting the myelopoetic dysregulation observed in severe COVID-19 cases. Epigenetic reprogramming of monocytes leads to rapid production of proinflammatory cytokines (IFNγ, TNF-α, and IL-1β) that can fight against the reduced T cell and dendritic cell function associated with COVID-19 fatalities. Such a rapid proinflammatory response has been proposed to be protective through defeating potentially severe infections before they can overwhelm the immune system and result in severe disease caused excessive systemic cytokine production. BCG has also been shown to enhance the effects of other, disease-specific, vaccines through mechanisms such as “bystander activation”, and moderate levels of epitope similarity between BCG and SARS-CoV-2 have been proposed to generate cross-reactive T cells.

The combination of increased mechanistic understanding of BCG’s heterologous immune priming effects and a growing body of research on COVID-19 disease pathology begins to suggest a model for how BCG vaccination may help in protecting from severe COVID-19 (Figure 1). Literature specifically addressing the effectiveness of BCG at improving morbidity and mortality resulting from COVID-19 disease remains varied and sometimes inconsistent [75]. This area of investigation is rapidly emerging and has garnered a great deal of interest [76,77,78]. Here, we review studies specifically investigating the effects of BCG vaccination on COVID-19 spread, morbidity, and mortality, excluding papers that have not yet been peer reviewed (Table 2).

Early in the pandemic, there was a great deal of discussion of the potential of BCG to reduce the mortality and morbidity stemming from COVID-19. O’Neill and Netea proposed the concept, but emphasized the need for rigorous randomized clinical trials, calling light to an issue where the lack of such properly controlled and well-designed trials has plagued similar research on BCG [79]. Desouky’s review proposed that a “cheap, licensed and readily available vaccine can be a tool to boost population immunity while waiting for a specific vaccine to come into the light” [80]. However, drawing conclusions from the limited literature where confounding variables are abundant and double-blinded studies are rare is difficult, especially when many of the most powerful BCG studies are still in progress. Fu et al. created a model based on country-level COVID-19 data from the Johns Hopkins University Coronavirus Resource Center to investigate the effect of BCG vaccination during the pandemic [81]. They created three models, with the first model estimating how a combination of both current and past BCG vaccinations per country affected the number of cumulative COVID-19 deaths per day, the second model estimating the impact of BCG vaccination in younger (<50) and older (50+) populations, expecting that people born after the 1970s WHO BCG vaccination programs would be more likely to be vaccinated, and the third model using the average BCG vaccination coverage between 1990 and 2018. Model 1 identified that, compared to countries with no BCG vaccination programs, countries with active BCG vaccination programs displayed a lower number of cumulative deaths early in the epidemic, though the effect was not statistically significant. In contrast, countries that previously had a BCG vaccination program but do not any longer appeared similar to countries with no BCG program. Relative to the first month following the first COVID-19 death in each country, the under-50 populations in high-BCG-coverage countries showed significantly fewer deaths during days 31–60, while no significant effect was observed in the over-50 population. Countries with higher BCG coverage displayed a significantly slower rate of increase in deaths, though the increase in protection was no longer significant after the third month. Interestingly, high BCG coverage reduced COVID-19 related deaths by 43.8% between days 31 and 90 following the first COVID-19 death. They concluded that there is evidence that high coverage with BCG vaccine in a country provides a protective effect for COVID-19 in the initial months of the epidemic in that country. However, when applying a “government response index” or a policy stringency index to identify how national epidemic responses interact with BCG vaccination, they found that a higher government response index offsets the effect of BCG on mortality over all time intervals, suggesting that the effect of BCG vaccination is reduced once government epidemic response policies are in place.

Much of the literature regarding the potential of BCG to help combat the COVID-19 pandemic examined relative mortality rates in similar countries with and without scheduled BCG vaccination. Berg et al. compared the initial 30 days of the COVID-19 epidemic in countries with mandatory BCG vaccination programs until the year 2000 against countries lacking BCG programs in that period [82]. They found the rate of increase in COVID-19 cases was significantly slower in countries with mandated BCG vaccinations, even when countries were weighted by case reporting quality. Countries with BCG vaccination programs also had a significantly slower growth rate of COVID-19-related deaths than those without mandatory BCG vaccinations. Overall, these data suggest that BCG vaccination contributed to “flattening of the curve in the spread of COVID-19”. Chimoyi et al. explored a similar line of investigation, adjusting for population size, gross domestic product, proportion aged over 65 years, stringency level measures, testing levels, and time difference [83]. Interestingly, no correlation between national BCG vaccination policy history and SARS-CoV-2 cases or deaths was observed, which they attributed to a weakening association between BCG vaccination and a beneficial effect as the national epidemics progressed. Wickramasinghe et al. investigated parameters indicating national BCG vaccination coverage and effectiveness, and their effect on the morbidity and mortality of COVID-19, finding that COVID-19 deaths were higher in countries with BCG coverage less than 95% when compared with countries with greater than 95% coverage, and that the presence of a universal BCG vaccination policy reduced cases and deaths from COVID-19 [84]. Senoo et al. examined the correlation between BCG vaccination and COVID-19 disease in 35 OECD countries, plus China and Taiwan, and found significantly lower COVID-19 infection and mortality rates in countries with current BCG vaccination programs when compared to countries that had a BCG vaccination program in the past, or never had BCG vaccination [85]. While this study found that BCG coverage did not correlate with COVID-19 cases or deaths, BCG coverage showed a moderate reduction of cases and deaths in high-income countries when stratified by income level. The authors noted a reduction in the COVID-19 test positivity rate as the percentage of the population that was tested increased, and suggested that a lag between cases and deaths may limit the ability to make comparisons between high and low income countries. Avoiding this problem, Ebina-Shibuya et al. looked at population-based COVID-19 mortality rates from only high-income countries, and found that rates were higher in countries that had never recommended BCG vaccination than those that currently recommend it [86]. Seven of the eight countries examined by Miyasaka et al. with very low total COVID-19 deaths have mandatory BCG vaccination, while those without widely administered BCG vaccination or with BCG programs that were discontinued over 20 years prior showed higher death rates [87]. Ozdemir et al. found that the COVID-19 case rate was significantly lower in BCG-vaccinated countries than in BCG-non-vaccinated countries, and the death rate was also significantly lower, as was the ratio of COVID-19 deaths per case [88]. Among European countries, the COVID-19 case rate and deaths per population and per case rate was lower in BCG-vaccinated countries. A meta-regression on data from 160 countries found that countries with greater than 70% BCG vaccine coverage reported 10.1 per 10,000 population fewer COVID-19 cases and countries with less than 70% coverage reported 6.5 per 10,000 fewer than non-vaccinating countries [89]. Countries with and without BCG vaccination policies were compared and found to have an COVID-19 fatality odds ratio of 0.15 (CI95: 0.14–0.16) for countries with BCG vaccination [90]. An investigation of the effect of BCG vaccination on elderly death rates when comparing countries that utilized BCG vaccination in the 1950s to countries that did not found no effect on COVID-19 case fatality rate or number of deaths per population, but since the strength of protection from BCG vaccination decreases over time, this result is not in conflict with the other studies supporting an impact of BCG on severity of COVID-19 [91].

It has been suggested that, due to confounding variables, not enough evidence is available to support or deny the hypothesis that BCG vaccination reduces COVID-19 incidence and mortality [92]. Using the measure of deaths per case/days of the epidemic instead of deaths per population, no significant correlations between the year of the establishment of universal BCG vaccination and mortality rates are observed [93]. An early review of the literature where 12 of the 13 papers were in pre-peer reviewed status found that confounding factors—such as demographics—were poorly addressed, and that results were conflicting [94]. Confounding factors such as the stage of the COVID-19 epidemic, development, rurality, population density, and age structure can impact observations and should be controlled for whenever possible [95]. Socially similar European countries display a strong correlation between Escobar et al.’s “BCG index”, an estimation of the degree of universal BCG vaccination deployment in a country, and reduction of COVID-19 mortality. While controlling for confounding variables reduced the significance of the association between BCG and reduction of COVID-19 deaths, it remained significant for several comparisons that controlled for social conditions and stage of the epidemic. States in the U.S. without BCG vaccination were found to exhibit significantly higher COVID-19 mortality rates than from states in countries that deploy BCG. Furthermore, significant correlation is observed between BCG index and reduction of COVID-19 mortality in European countries administering BCG to infants. After the COVID-19 pandemic substantially shifted toward South America by August 2020, the negative correlation of BCG vaccination to COVID-19 mortality rate was no longer significant [96]. Marín-Hernández et al. drew on the Escobar study and the BCG vaccination campaign in West Germany, and used modeling to project a corresponding median infection rate decrease of 40% and a 37% reduction in median mortality [97]. Comparison of COVID-19 rates and death rates in East and West Germany produces results inconsistent with BCG providing protection, even among age groups who would have been expected to show differences due to differing BCG vaccination policies in East and West Germany during their infancy, but once again, waning immunity over time could well be responsible [98]. In the top 61 countries with the highest median age, COVID-19 death rates inversely correlate with the country’s BCG vaccination rate [99]. A multivariable analysis of COVID-19 death and case rates found a strong negative correlation between those rates and years of BCG administration and found the strongest effect to be in the youngest age group [100]. Unsupervised machine learning found that COVID-19 death rates were lower in countries that had a BCG vaccination policy for the previous 15 years, including in younger populations, but the same effect was not seen for other vaccination programs [101]. A total of 61 factors—including BCG vaccine coverage, percentage of urban population, and percentage with insufficient physical activity—were examined in 173 countries, using morbidity and mortality as outcomes, and found that recent BCG vaccine coverage reduced COVID-19 mortality, but not morbidity [102]. A pooled analysis of countries with and without BCG vaccination policies found a mortality rate of 1.31% for countries with BCG vaccination versus 3.25% for those without, resulting in an absolute risk reduction where one death can be prevented if 52 individuals are vaccinated [103]. Analysis of the impact of BCG on COVID-19 spread rate and mortality found that universal BCG vaccination policies correlate with lower COVID-19 spread rates [104]. However, BCG vaccination no longer correlated with COVID-19 spread rate when only countries with high COVID-19 testing were considered. Percent COVID-19 mortality was significantly lower in countries with universal BCG vaccination, but that association is lost once age is controlled for. It has been proposed that TB infection, particularly latent TB, may also provide non-specific immune priming that could protect against COVID-19, and thus as countries with higher rates of past TB incidence demonstrate lower rates of COVID-19 mortality (rate ratio of COVID-19 mortality of 2.70 per 1 unit decrease in past TB incidence rate, CI95: 1.09–3.30), recent TB rates should also be considered as a confounding variable when assessing BCG’s effects on COVID-19 [105].

A study comparing COVID-19 infection rates for Italian physicians failed to find a significant difference between those vaccinated with BCG and not vaccinated [106]. The connection between COVID-19 severity and *Mtb* exposure or BCG vaccination in Turkish healthcare workers was examined, finding that they were both associated with increased hospitalization and observation of radiological infiltrates in healthcare workers with COVID-19 [107]. In Italy overall, BCG vaccination was protective against the risk of severe COVID-19, with a BCG odds ratio of 0.47 (CI95: 0.22–0.98), but the protection was not evident for healthcare workers [108]. In California, an observational study of healthcare workers found that BCG vaccination, but not other vaccinations, was associated with both lower anti-SARS-CoV-2 IgG seroprevalence and self-reported COVID-19 symptoms [109].

With the difficulty of controlling for so many variables when comparing multiple countries in a single study, studies that cover only a single country or region may be more meaningful. One retrospective observational study where BCG vaccination showed promise examined a population of 120 consecutive adult and primarily Latino/Hispanic COVID-19 patients at a healthcare center in Providence, Rhode Island in March and April 2020 [110]. The median age was 39.5 years with the non-BCG-vaccinated group having a median age of 31.0 years versus the BCG-vaccinated group’s median age of 41.0 years. Despite the presumptive length of time between vaccination and development of COVID-19, the non-vaccinated group was over twice as likely to be referred to the emergency room, and was over four times as likely to be hospitalized. However, it should be noted that while two of the hospitalized non-vaccinated patients had a COPD or asthma comorbidity, none of the hospitalized patients with BCG vaccination did. Furthermore, the non-vaccinated group in this study was also significantly more likely to have those comorbidities. Even after adjusting for comorbidities, the beneficial effects of BCG vaccination remained. Symptoms were similar for both groups, though the vaccinated group was more likely to display myalgia [110]. Comparison of COVID-19 infection rates and proportions for subjects born during the three years before and after cessation of Israel’s inclusion of BCG in the national immunization program found no difference in the proportion of positive test results or positivity rates per 100,000 [111]. Newborn BCG vaccination was discontinued in Sweden in 1975, allowing comparison of COVID-19 case rates for Swedes born in the year before and after, and the lack of difference in rates allowed them to reject their hypothesis that universal BCG vaccination reduces the number of COVID-19 cases by 19% and the number of hospitalizations by 25% [112]. BCG vaccination coverage and COVID-19 were examined in Ecuador, and a higher prevalence of cases for people in the 50 to 64-year-old group was found than in the 20 to 49 group, and areas with low BCG coverage since 1980 showed a high prevalence of COVID-19 in the 20 to 49 group [113]. A study of Taiwanese children born after 1985 failed to find any reduction in COVID-19 severity based on BCG vaccination status, but the study was limited by very low population numbers [114]. BCG vaccine coverage in five Japanese prefectures with no COVID-19 infections was significantly higher than that in five prefectures with a high prevalence of infections, leading the authors to propose that that infant BCG vaccination has a protective effect against mass infection [115]. A retrospective cross-sectional study in Istanbul between March and June 2020 of patients diagnosed with COVID-19 pneumonia found that, while the case rate was lower for those with BCG vaccination, it was not associated with disease severity [116]. In a retrospective study of bladder cancer patients who received BCG therapy as compared to the same number of urology patients diagnosed with low-risk bladder cancer and not receiving BCG, the number of subjects who were identified to be COVID-19 positive was not significantly different [117]. A study where staff at the Emirates International Hospital were offered a booster BCG vaccine in early March 2020 and then tested for COVID-19 in June of 2020 found that 8.6% of the non-booster vaccine group tested positive for COVID-19, while none of the BCG booster group tested positive [118]. Randomization and controls for demographic, work role, and other confounding factors would have strengthened this study, but the data suggest a beneficial effect of BCG vaccination. Based on these observations, more controlled studies are needed to reconcile the various results of the many environmental/ecological studies. 

At present, transcriptomic data provides evidence for the potential of BCG vaccination to fight COVID-19 and that it can induce long-term changes in the blood immune cell transcriptome that partially mimic and partly oppose transcriptomic changes induced by viral respiratory illnesses, including COVID-19 [119]. Upregulation of viral defense genes is observed. It is intriguing that myeloid cell activation genes are downregulated in vaccinated subjects, but upregulated in COVID-19 patients. Microarray datasets for normal human bronchial epithelial cells from a SARS-CoV-2 infected person and RNAseq data from BCG-vaccinated persons before and after vaccination display upregulation in 45 distinct KEGG pathways for SARS-CoV-2 infection that overlap with pathways downregulated following the BCG vaccination [120]. Most notably, these included IL-17 and NLR pathways that are downregulated by BCG, suggesting that BCG vaccination could fight acute respiratory distress syndrome in COVID-19 patients.

## 7. BCG Trials for Protection against COVID-19 Currently in Progress

Numerous trials investigating the potential of BCG vaccination to fight the COVID-19 pandemic are currently underway, incorporating diverse strains of BCG and potentially providing further insight into optimal strain selection. Two prior reviews have cataloged these studies, and more up-to-date information for some trials can be accessed at https://clinicaltrials.gov/ (accessed on 1 September 2021) [121,122]. Here, we discuss studies BCG studies with COVID-19 as their primary disease that are currently listed as recruiting, not yet recruiting, and active. Europe has the majority of these studies, with The Netherlands having two studies enrolling the elderly and one for healthcare workers, all using the BCG Danish strain 1331. Denmark has studies for both healthcare workers and elderly subjects, using the Danish strain, similar to the French healthcare worker study. Poland has one healthcare worker study using the BCG-10 (Polish Moreau) strain and Germany has two studies, covering elderly and healthcare workers, using the listeriolysin-expressing VPM1002 rBCG strain [123]. In the U.S., a multi-site study in Texas and California utilizes the Tice BCG strain to investigate protection for healthcare workers, first-responders, essential workers, and members of high-risk groups. A Mexican study examines protection of healthcare workers using the Tokyo 172 strain and a Canadian study focused on essential workers uses VPM1002. Brazil hosts one study with general eligibility, and one for those with recent COVID-19. India hosts a study covering the elderly and using BCG from the Serum Institute of India; South Africa and Egypt host healthcare worker studies using Danish strain 1331; Australia hosts a healthcare worker study using Danish strain 1331; and a multi-site study of healthcare workers in Cape Verde, Guinea-Bissau, and Mozambique also uses the Danish strain. In contrast to the majority of studies currently focused on adult vaccination, one study to determine the effects of BCG vaccination in a low-mortality, high-income setting is the Melbourne Infant Study: BCG for Allergy and Infection Reduction [124].

## 8. BCG Strains: An Important Consideration for Immune Priming?

One explanation that has been proposed for the variability of BCG vaccine trials is that different strains of BCG may confer different levels of immunity, possibly due to the number of passages that strains underwent prior to lyophilization [13]. Multiple BCG strains are used globally, with some countries utilizing only a single strain, and other countries utilizing multiple strains (Table 3) [125]. A 1983 study found over a 50-fold difference in viability between lyophilized strains, with the Japanese strain showing the highest viability [126]. However, when inoculated into mice, observations of delayed local reactions, kinetics of TST, spleen indices, and total CFU cultured from organs showed little evidence for significant differences in strains. A more recent study compared five BCG strains commonly used for BCG vaccine production (Glaxo 1077, Japanese 172, Pasteur 1173P2, Prague, and Russian) [127]. They found that the Japanese and Prague strains exhibit lower growth in BALB/c mice than the Pasteur, Russian, and Glaxo strains. Japanese and Prague strains also failed to suppress subsequent infection by a recombinant Pasteur BCG strain [127]. However, the results of their attempt to quantify differences in immune responses by proliferation of cells drained from lymph nodes upon response to PPD, as well as IFNγ and IL-2 production upon stimulation, did not consistently correlate with observed in vivo protection. Lymph node cells from mice vaccinated with the Pasteur and Russian strains demonstrated higher levels of specific lysis of infected bronchoalveolar macrophages, and BCG Japan demonstrated significantly lower IgM and IgG antibody titers. A 2017 meta-analysis of BCG for treating of non-muscle invasive bladder cancer found that when comparing the Tokyo, Pasteur, and TICE strains, none were significantly better than the others at preventing recurrence [128]. A 2016 study infected SCID mice and found that the Phipps, Frappier, Pasteur, and Tice strains resulted in mortality of these immunocompromised mice, while the Japan, Birkhaug, Sweden, Glaxo, and Prague strains did not [129]. When those strains were used to immunize BALB/c mice, the Phipps, Frappier, Pasteur, Tice, Danish, Prague, Russia, and Moreau strains had significantly lower *Mtb* burdens after challenge than the non-vaccinated mice. In contrast, those vaccinated with the China, Glaxo, Japan, Birkhaug, and Sweden strains did not show protection. Additionally, those vaccinated with the Pasteur, Tice, or Danish strains exhibited the lowest *Mtb* burden in their spleens. It has been suggested that the more virulent BCG strains are the most protective when used as vaccines. BCG strains have been grouped on the genetic basis of tandem duplication (DU2), with DU2 group IV containing the Phipps, Pasteur, Frappier, and Tice strains being the most virulent [129]. Defects in phthiocerol dimycocerosate and phenolic glycolipid synthesis may be responsible for the low virulence of the BCG Glaxo strain [130]. Similarly, a frameshift mutation in *phoP* in BCG Prague may explain the low virulence in this strain [130]. In addition to established naturally passaged strains, BCG strains that express the ESX-1 type VII secretion system found within the RD1 region of *Mtb*, but not BCG, and responsible for secretion of virulence factor ESAT-6, could potentially increase protection against *Mtb* infection, though the question of whether such enhanced protection may extend to non-specific effects remains unclear [131]. However, it is also possible that expression of this region could increase virulence of BCG to the point that higher frequencies of side-effects would be observed.

One study found that there was no difference in immunogenicity between the Danish-SSI 1331 and Glaxo-Evans 1077 strains based on IFNγ and conversion rates, though they did find that the Danish strain resulted in smaller scar sizes [132]. A randomized trial with 164 infants suggested several immunological differences [133]. The Denmark and Japan strains induce significantly higher polyfunctional CD4+ T cells than the Russia strain. Additionally, those that received the Denmark strain had higher proportions of CD107-expressing cytotoxic CD4+ T cells, with CD107 being used as a measure of cytotoxic capacity, and those who were immunized with the Japan strain displayed higher concentrations of secreted Th1 cytokines. Vaccination of infants using the Denmark and Brazil BCG strains preferentially induces Th1-associated cytokines involved in adaptive immunity, contrasting with the Japan strain, which preferentially induces proinflammatory-associated cytokines [134]. In contrast, several studies have failed to find a correlation between BCG vaccine strain and the immune response [15,18,135]. Interestingly, comparing 162,953 newborns receiving BCG vaccination in a randomized trial in Hong Kong, fewer persons who received the more virulent Pasteur strain developed TB in a four-year period than those who received the less virulent Glaxo strain (3.43 vs. 4.55 per 10,000) [136]. On the other hand, a retrospective study examining vaccinated children in Kazakhstan found that the relatively low-virulence Japan strain was the most effective at preventing clinically notified TB and culture-confirmed TB (69% and 92%, respectively), contrasting with the relatively virulent Russian strain at 22% and 51% effectiveness. These strains were employed over different time periods, with the Japan strain being used most recently, introducing many potentially confounding variables. Looking at infants in Uganda who received Denmark, Russia, or Bulgaria strains at birth, the Denmark strain produced significantly more scarring and those infants with scars displayed stronger IFNγ and IL-13 responses, though no strain-dependent differences in mortality were observed, and non-specific cytokine responses were not associated with scarring [137]. Growth conditions of the vaccine strain plays an important role in the strength of the vaccination, as observed by one study where “slow growth” BCG lots were found to produce positive PPD responses and induce higher cytokine responses in a higher percentage of subjects than normal lots. Specific characteristics of the growth medium composition—in this case, a change in glycerol supplier, or growth conditions—greatly influence gene regulation and, thus, virulence and effectiveness of the vaccine lot [42]. 

## 9. Obstacles to BCG Implementation and Ways around Them

The U.S. has previously resisted implementation of scheduled BCG vaccination. The BCG vaccine may be especially beneficial in the U.S. and other high-income countries where exposure to environmental mycobacteria is rare. School children in England exhibit a low level of IFNγ response to environmental mycobacteria PPD, but that response is increased after BCG vaccination. This is in contrast to children in Malawi, who exhibited a strong response to mycobacterial PPD prior to BCG vaccination, and display only a small increase in their response after receiving BCG [138,139,140]. As evidence mounts that BCG vaccination could aid in the ability of the U.S. population to combat future respiratory disease pandemics, the question remains what obstacles implementation of large-scale BCG vaccination in the U.S. could face, and how those obstacles could be overcome.

Safety of BCG vaccination is easily addressed, since over 4 billion administrations have been performed globally and serious adverse events in non-HIV-positive individuals are extremely rare [141]. Complications following BCG injection do not normally occur when immunization is performed following recommendations [3]. Per WHO guidelines, BCG vaccination is contraindicated for persons with impaired immunity, symptomatic HIV infection, known or suspected congenital immunodeficiency, leukemia, lymphoma, or generalized malignant disease, for patients under immunosupressive treatment, corticosteroids, alkylating agents, antimetabolites, or radiation, and in pregnancy [3]. Overdoses can also be clinically managed. In one case, a 14-year-old girl received 10 times the intended dose, and exhibited a tender lump at the injection site that was treated with excision followed by isoniazid and rifampicin for six weeks, leading to no complications [142].

The objection has been raised that increased deployment of BCG could jeopardize the continued supply of the vaccine to higher risk areas where it is routinely deployed in infants, and thus BCG should only be used in limited clinical trials rather than being used for community protection from COVID-19 [143,144]. However, others argue that supply of BCG will ultimately be of less importance [80]. Implementation of BCG as a recommended vaccine would require scaling-up of manufacturing facilities or approval of an overseas-produced vaccine, since currently only one manufacturer produces BCG licensed for use in the U.S. [145]. Sanofi previously also produced BCG at a facility in Canada for bladder cancer therapy use in North America and Europe, but ceased production in 2012 due to a flood-related mold contamination issue, before finally announcing that Sanofi was exiting the market in 2017 [146]. Likewise in 2014, the only facility in the U.S. experienced a contamination issue with a batch of Tice strain BCG, creating a supply shortage that took 6 to 12 months to alleviate, partially due to the length of time required for growth of *M. bovis* [145]. The FDA does not view drug shortages as their primary concern, possibly increasing regulatory production requirements and restriction of entry by foreign drug products [145]. The global production and demand of BCG has been examined to better understand the individual issues responsible for supply shortages in previous years [147]. In 2017, the global supply of BCG vaccine was forecasted to be 1.5 times the global demand, largely benefiting from the number of global BCG suppliers. Two lessons are clear: the length of time required to ramp up BCG production indicates that BCG production capacity must be developed well ahead of demand if BCG vaccination is to be used to help fight the next global respiratory pandemic, and creating an economic incentive for overseas BCG producers to supply BCG as an FDA-approved pharmaceutical in the U.S. will improve the robustness of the BCG supply for scheduled vaccination. In January 2021, the only U.S. supplier announced the construction of a new facility to expand their manufacturing capability for TICE BCG. Continued investment in BCG production suggests that larger scale of BCG vaccination trials in the U.S. would not interfere with existing therapeutic uses [148]. Additional supply may also allow the cost of BCG vaccines in the U.S. to be brought more in line with those paid for BCG vaccines elsewhere. In 2020, the cost per dose of BCG vaccine to UNICEF, the largest BCG vaccine purchaser, was US$0.12 per dose, compared to $0.22 for self-procurement for middle-income countries, making BCG one of the lowest-cost vaccines [149]. In comparison, TICE BCG currently costs US$152.80 per vial or effectively $0.31 per intradermal dose, since there are 500 doses in each vial, nearly 30% higher than from UNICEF [150].

The BCG scar may be an important consideration, both for public acceptance of large-scale BCG vaccination and as an indicator for effectiveness. In Guinea-Bissau, children who were participating in a measles vaccine trial were examined for a BCG scar at 6 months of age, and those who had been BCG-vaccinated were given a TST [151]. Children with a BCG scar had significantly lower mortality compared to those without; in the first 12 months of follow-up, the mortality ratio was 0.41 (CI95: 0.25–0.67). Of those with scars, those who also demonstrated a positive TST had a mortality ratio of 0.45 (CI95: 0.24–0.85) compared to those who were TST negative. In a more recent trial in Guinea-Bissau, low and normal birthweight infants received BCG at birth, and mortality was evaluated at one year [152]. The low birthweight cohort demonstrated an adjusted mortality rate ratio 0.42 (CI95 0.19–0.93) for those with a BCG scar versus those without. For the low birthweight children who had both a positive TST reaction and a scar, the ratio improved to 0.22 (CI95: 0.05–0.87). Review of six studies found that subjects displaying a BCG scar showed a mortality rate ratio of 0.61 (CI95: 0.51–0.74) versus those without [153]. The percentage of subjects that displayed a BCG scar varied from 52% to 93%. It should be noted that viremia after vaccination by an attenuated yellow fever strain did not show correlation with heterologous T cell responses, including IFNγ production, which suggests that in adults or high-wealth countries, lack of a BCG scar may not necessarily indicate a reduction in non-specific protection [43]. When children in Guinea-Bissau with and without a BCG scar were assessed at six months of age and again 12 months later, the group with the BCG scar demonstrated a mortality ratio of 0.45 (CI95: 0.21–0.96) [154]. Interestingly, there were no differences in causes of death, but there was a reduction in death from malaria in the scar group. “Vaccine hesitancy” represents a concern for deployment of vaccines [155]. The recent “5C model of the drivers of vaccine hesitancy” offers five person-level concerns that drive vaccine hesitancy: confidence, complacency, convenience (or constraints), risk calculation, and collective responsibility [156]. While the “safety” component of the confidence and risk calculation “C’s” can be addressed by a century of safety data, the risk calculation component may be affected by BCG vaccination’s likelihood of producing a scar at the injection site. A 1993 survey of British high-school children found that 35% of girls and 7.8% of boys found their BCG scars unacceptable, expressing a desire for less visible injection sites [157]. In consideration of whether BCG strain choice can reduce prevalence of BCG scarring and increase acceptance of BCG vaccination, considered independently of strain effectiveness, when the Danish and Russian BCG strains were compared in Guinea-Bissau, children vaccinated with Danish BCG were more likely to develop a scar than children vaccinated with the Russian strain (97% vs. 87%), though such a small difference is unlikely to make a practical difference in vaccine acceptance [158].

The route of BCG administration may be a key consideration as well. Intradermal administration is preferred due to increased reliability of stimulating a skin test response as well as a Th1-associated cytokine response [159,160,161,162]. Another study compared the Danish strain administered intradermally with the Japan 172 strain administered intradermally or percutaneously [163]. The Japan strain introduced percutaneously produced greater secretion of Th1-type cytokines as well as higher frequencies of IFNγ-producing T cells than the intradermal Danish BCG, as well as a smaller improvement over the Japan strain introduced intradermally. In contrast, an earlier study that found intradermal inoculation produced significantly stronger lymphoproliferative and IFNγ responses compared to percutaneous inoculation in adults [164]. Other than the age difference in the studies, another factor to consider with percutaneously administered BCG appears to be ensuring sufficient numbers of epidermal needle punctures, as noted by a study that found that frequency of proliferating CD4+ T cells after BCG stimulation at six days correlated positively with the number of punctures [165]. However, the lack of scarring with percutaneous administration may offer an option that reduces public opposition to adding BCG vaccination to the U.S. vaccine schedule [166,167]. A South African trial covering 11,680 infants receiving the Tokyo 172 BCG strain at birth who were then followed for two years found no significant difference in TB rates between those who were inoculated via percutaneous versus intradermal routes, suggesting that percutaneous administration could be considered in order to improve public acceptance of BCG in high income countries [168].

Future developments may make BCG vaccination safer or more effective. One replication-limited strain of BCG constructed on a Tice background, rBCG(mbtB)30, showed improved protection relative to BCG in a Guinea pig *Mtb* challenge model as well as being more attenuated in SCID mice [169]. Another study showed that a BCG strain producing the membrane-perforating listeriolysin (Hly) of *Listeria monocytogenes* showed greater protection against *Mtb* challenge in BALB/c mice [170]. There are a number of other recombinant BCG vaccine efforts underway [171].

Another objection to BCG vaccination has been that it renders the inexpensive TST test ineffective. However, the fact that Interferon Gamma Release Assays (IGRAs) are not compromised by BCG vaccination eliminates this concern and should allow monitoring TB exposure in the U.S. even if a national BCG vaccination program is initiated [172]. Two IGRAs are FDA approved and commercially available in the US: the QuantiFERON-TB Gold In-Tube test (QFT-GIT) and the T-SPOT.*TB* test [173]. While IGRA testing is more expensive than TST, the price difference is small in the context of TB control programs (US$35–40 per IGRA test versus $3–13 per TST), and programs can benefit by having a single test that works both on those born in the U.S., and those born in areas where BCG vaccination is common [174]. Furthermore, individuals only require a single healthcare visit for an IGRA test, whereas TST requires two—one for delivery of the PPD and the second for examination of the response a few days later—making the economic cost most likely higher and the cost in terms of patient time greater for TST.

## 10. Conclusions

Increasing support for a U.S. BCG vaccination program will likely require release of the data from ongoing trials and possibly additional clinical trials. The cost versus overall benefit of BCG vaccination in the U.S. should be carefully considered and, in light of new diagnostic assays that allow TB monitoring in the presence of BCG vaccination and the exceptional safety profile of BCG, it is time to re-visit whether the potential benefits are greater than any risks. Is BCG vaccination cost-effective in a developed, high-income country? The Irish neonatal BCG vaccination program considered this question, but did so exclusively in terms of TB-related effects, with their model finding that a universal BCG vaccination program costs €204,373 per life year gained (LYG) and a selective BCG vaccination programs costs €143,233 per LYG [5,175]. In this context, a selective strategy would cost €1,055,692 less per 4.8 life years lost per birth cohort. However, this type of analysis only considers the TB-related benefits of BCG vaccination. Once non-TB burdens are considered, the question changes dramatically. For example, murine studies suggest that BCG can enhance efficacy of other specific vaccines. BCG vaccination plus a BCG booster two days prior to influenza A challenge improved survival [176]. BCG vaccination significantly improves antibody titers in a hemagglutination inhibition assay against an influenza A strain related to one used in the trivalent seasonal influenza vaccine, though significant titer differences were not observed for the other influenza A strain or influenza B tested, possibly due to the very small size of the study and already high baseline titers [177]. Enhancement of specific vaccines is just one example of the potential non-specific beneficial effects that BCG vaccination may provide. Protection from respiratory infections, allergies and autoimmune disorders, and even diabetes and cancer have been associated with BCG vaccination. With the cost of BCG vaccination being less than a dollar per person, even modest benefits in each area would make BCG vaccination cost-effective for the U.S. overall, particularly considering the currently extremely high rates of diabetes and cancer in the U.S. These long-term benefits to human health stand to dwarf the minimal economic burden of implementing recommended BCG vaccination and conversion of all TB surveillance to an IGRA-based strategy. Even moderate relief of overstressed hospitals thanks to lower ICU utilization rates during the next pandemic, especially in the early days of the pandemic while the government epidemic response is still ramping up, is another significant benefit that BCG vaccination could provide.

Deployment and expansion of well-designed, well-controlled (including placebo-controlled) experimental trials to examine non-specific effects in ways that can provide definitive conclusions will provide the next step in exploration of the strength of BCG vaccinations’ broad protective effects that existing studies have not yet fully provided. Trials including different BCG strains may also be needed to optimize the strain used for specific applications, such as high dose treatment for bladder cancer or immune training to mitigate severity of viral infections. These studies could be deployed alongside regional roll-outs of recommended BCG vaccination, as was done in England [11]. Importantly, it has been shown that BCG can be safely used during the COVID-19 pandemic, having been shown not to increase hospitalization, sickness, or self-reported symptoms, regardless of the strength of the BCG response, making further examination warranted in light of newly available TB exposure monitoring methods that are not impacted by BCG vaccination [178]. Arguably, the clearly demonstrated absence of harm and outstanding safety profile of BCG—even in infants, with the potential for significant long-term health benefits—makes careful re-examination of our national BCG vaccination policy a critical next step toward improving U.S. vaccination policies.

## Figures and Tables

**Table 1:** Overview of selected literature on immuno-modulatory and trained immunity effects of BCG.

Publication	Summary	Organism
Andersen et al., 2013	BCG re-vaccination skews the response to PPD towards a Th1 direction, with a greater IFNγ/IL-13 ratio.	Human
Arts et al., 2015	Monocytes from subjects with complete NOD2 deficiency failed to demonstrate trained immunity.	Human
Arts et al., 2018	BCG vaccination produces epigenetic reprogramming of monocytes, and reduces viremia when challenged with the yellow fever vaccine.	Human
Eggenhuizen et al., 2021	BCG protein sequences with homology to SARS-CoV-2 proteins induce enhanced CD4+ and CD8+ T cell responses to SARS-CoV-2 antigens.	Human
Jensen et al., 2015	Infant BCG vaccination enhanced cytokine responses (IL-1β, IL-6, TNF-α, and IFNγ) to heterologous stimulation.	Human
Kaufmann et al., 2018	BCG vaccination affects long-lived cells in the bone marrow and promotes polarization of multipotent progenitor cells towards myelopoiesis at the expense of lymphopoiesis.	Mouse
Kleinnijenhuis et al., 2012	BCG vaccination produces an enhanced cytokine response to heterologous stimulation, mediated by NOD2.	Human
Kleinnijenhuis et al., 2014	Heterologous Th1 responses remain strong for a year following BCG vaccination.	Human
Kleinnijenhuis et al., 2014	BCG vaccination enhances proinflammatory cytokine response to heterologous stimulation.	Human and Mouse
Mathurin et al., 2009	BCG-vaccinated mice’s resistance to vaccinia virus infection was removed by depleting CD4+ T cell populations, suggesting both the innate and adaptive immune components play a role in trained immunity.	Mouse
Ugolini et al., 2018	As a live vaccine, BCG can activate the TLR8 bacterial RNA-detecting PAMP, which regulates T_FH_ cell differentiation and downstream cytokine responses.	Human
Urban et al., 2020	Similarity of T cell and B cell epitopes in the BCG Pasteur strain’s proteome with SARS-CoV-2 peptides may create antigenic cross-reactivity, incorporating adaptive immune response into trained immunity for COVID-19.	Human
Uthayakumar et al., 2018	“Bystander activation” of polyclonal effector T cells by BCG vaccination may reduce their threshold for response when challenged again by heterologous infections.	N/A
Wen et al., 2008	BCG vaccination enhances trimethylation at promoters associated with increased transcription of proinflammatory cytokine genes.	Mouse

**Table 2:** Conclusions of literature on BCG and COVID-19.

Study Type	Positive Conclusion	Negative Conclusion
Ecological Study	20	12
Modeling	2	-
Literature Review	-	2
Retrospective Observational	1	-
Immunological Research	3	-

Conclusions drawn by COVID-19-specific BCG studies. Studies were determined to have a positive conclusion if they determined that BCG vaccination slows the rate of epidemic spread or decreases hospitalizations, disease severity, or mortality for COVID-19 for at least one demographic group at one stage in the pandemic.

**Table 3:** BCG strains currently used in selected countries.

Danish SSI	BCG Japan/Tokyo 172	Pasteur	Moscow/SII	Bulgaria	Other
Afghanistan	Bhutan	Argentina	Bangladesh	Bulgaria	Angola (Connaught)
Belarus	Canada	Belgium	Gambia	Estonia	Australia (Connaught)
Czech Republic	Finland	Bosnia and Herzegovina	India	Hungary	Brazil (Moreau)
Denmark	Ghana	Colombia	Mongolia	Nigeria	China (various)
Ethiopia	Japan	El Salvador	Russian Fed.	Slovak Rep.	Congo (various)
France	Kazakhstan	Indonesia	Togo	Ukraine	Croatia (Moreau)
Germany	Kuwait	Iran	Turkey		Moldova (Japan and India)
Greece	Malaysia	Montenegro			Peru (Meriuex)
Greenland	Marshall Islands	Republic of Korea			Poland (Moreau)
Ireland	Mexico	Senegal			Saudi Arabia (various)
Italy	Pakistan	Serbia			Uganda (various)
Latvia	Philippines	Tunisia			
Malta	Portugal	Uruguay			
Netherlands	Taiwan				
New Zealand	Tajikistan				
Norway	Thailand				
Romania					
Singapore					
Slovenia					
South Africa					
Sudan					
Sweden					
Switzerland					
United Kingdom					

BCG strains used by each country. Data from the BCG World Atlas (http://www.bcgatlas.org/, accessed on 1 September 2021).
